# Pooled Analysis of Bleeding, Major Adverse Cardiovascular Events, and All‐Cause Mortality in Clinical Trials of Time‐Constrained Dual‐Antiplatelet Therapy After Percutaneous Coronary Intervention

**DOI:** 10.1161/JAHA.120.017109

**Published:** 2020-08-11

**Authors:** John D. McClure, Jennifer C. Ramsay, Colin Berry

**Affiliations:** ^1^ British Heart Foundation Glasgow Cardiovascular Research Centre Institute of Cardiovascular and Medical Sciences University of Glasgow United Kingdom; ^2^ West of Scotland Heart and Lung Centre Golden Jubilee National Hospital Clydebank United Kingdom

**Keywords:** acute coronary syndrome, antiplatelet agent, dual antiplatelet therapy, meta-analysis, percutaneous coronary intervention, Acute Coronary Syndromes, Thrombosis, Treatment, Percutaneous Coronary Intervention

## Abstract

**Background:**

The net clinical benefit of dual antiplatelet therapy (DAPT) reflects the paradoxical effects of an increased risk of bleeding and a reduced risk of major adverse cardiovascular events. A time‐constrained approach to DAPT has been recently investigated in 5 multicenter trials including GLOBAL LEADERS, STOPDAPT2 (Short and Optimal Duration of Dual Antiplatelet Therapy After Everolimus‐Eluting Cobalt‐Chromium Stent‐2), SMART‐CHOICE, TWILIGHT (Ticagrelor With Aspirin or Alone in High‐Risk Patients After Coronary Intervention), and TICO (Ticagrelor Monotherapy After 3 Months in the Patients Treated With New Generation Sirolimus Stent for Acute Coronary Syndrome).

**Methods and Results:**

We undertook a pooled analysis of these trials to assess the overall associations between time‐constrained P2Y12 inhibitor monotherapy (aspirin‐free regimen) for bleeding events, major adverse cardiovascular events, and all‐cause mortality as compared to standard care with DAPT for at least 12 months post‐percutaneous coronary intervention. We implemented a DerSimonian and Laird random effects meta‐analysis using the metafor package in R. 32 361 randomized trial participants, including 16 898 (52.2%) who had a history of acute coronary syndrome, underwent percutaneous coronary intervention, and had outcome data available. P2Y12 inhibitor monotherapy from 1 to 3 months was associated with a reduced risk for bleeding (hazard ratio [HR] 0.60; 95% CI, 0.45‐0.81), including in the acute coronary syndrome group in which the magnitude of risk reduction was greatest (HR 0.50; 95% CI, 0.41‐0.61). The estimates of the effect of P2Y12 inhibitor monotherapy on the HR were also favorable for major adverse cardiovascular events (0.88; 95% CI, 0.77‐1.02) and all‐cause mortality (0.85; 95% CI, 0.71‐1.03).

**Conclusions:**

Compared with DAPT for 12 months post‐percutaneous coronary intervention, P2Y12 inhibitor monotherapy from 1 to 3 months substantially reduces the risk of major and fatal bleeding and, in addition, confers potentially protective effects, for major adverse cardiovascular events and all‐cause mortality. Considering patient safety, the results support a strategy of DAPT for 1 to 3 months followed by aspirin‐free P2Y12 inhibitor monotherapy.

**Nonstandard Abbreviations and Acronyms**
ACS: acute coronary syndrome
DAPT: dual antiplatelet therapy
MACE: major adverse cardiovascular events
PCI: percutaneous coronary intervention

Aspirin is established as the first‐choice antiplatelet medication for secondary prevention of atherothrombotic events in patients with coronary artery disease.^1^, ^2^, ^3^ Because aspirin leads to incomplete antiplatelet inhibition, additional, dual antiplatelet therapy (DAPT) with an inhibitor of the platelet P2Y12 receptor is now evidence based.^2^, ^3^ In patients with stable coronary artery disease or an acute coronary syndrome (ACS) who are undergoing percutaneous coronary intervention (PCI), DAPT for 12 months is recommended in practice guidelines.^4^ After discontinuation of DAPT, the options for long‐term secondary prevention of atherothrombotic events include monotherapy with either aspirin or a P2Y12 inhibitor agent.

The CAPRIE (Clopidogrel versus Aspirin in Patients at Risk of Ischaemic Events) trial showed that in 19 185 patients with atherosclerotic disease, including 11 630 with myocardial infarction as the qualifying event, compared with aspirin monotherapy (325 mg per day), clopidogrel monotherapy (75 mg per day) for 1.91 years reduced the incidence of ischemic stroke, myocardial infarction, or vascular death by 8.7% (95% CI, 0.3–16.5; *P*=0.043).^5^ There was a treatment interaction by disease subgroup suggesting that the true benefit of clopidogrel was less certain in patients with myocardial infarction (7.4% [−5.2 to 18.6]). On the other hand, severe bleeding was more common with aspirin, notably severe gastrointestinal bleeding (2.66% versus 1.99%; *P*<0.05). A meta‐analysis of 9 randomized trials of P2Y12 inhibitor monotherapy or aspirin for secondary prevention involving 42 108 patients found a borderline risk reduction conferred by P2Y12 inhibitor for myocardial infarction (odds ratio, 0.81; 95% CI, 0.66–0.99) but no evidence of a between‐group difference in the risks of bleeding or all‐cause mortality.^6^ The ongoing ADAPTABLE trial aims to clarify the optimal dose of aspirin (81 or 325 mg, daily) for secondary prevention.^7^ To date, no trial has compared aspirin monotherapy versus P2Y12 inhibitor monotherapy after initial treatment with dual‐antiplatelet therapy for 1/3 months after myocardial infarction.

Antiplatelet therapy is associated with an increased risk of major and fatal bleeding. The net clinical benefit reflects the paradoxical effects of an increased risk of bleeding, a reduced risk of major adverse cardiovascular events (MACE) secondary to atherothrombosis, and, potentially, favorable effects on all‐cause mortality. The competing risks of these events vary over time and are greatest early after the initial PCI. Accordingly, a time‐constrained approach to DAPT after PCI has now been investigated in 5 multicenter clinical trials,^8^, ^9^, ^10^, ^11^, ^12^ including GLOBAL LEADERS,^8^ STOPDAPT‐2 (Short and Optimal Duration of Dual Antiplatelet Therapy After Everolimus‐Eluting Cobalt‐Chromium Stent‐2),^9^ SMART‐CHOICE,^10^ TWILIGHT (Ticagrelor With Aspirin or Alone in High‐Risk Patients After Coronary Intervention),^11^ and TICO (Ticagrelor Monotherapy After 3 Months in the Patients Treated With New Generation Sirolimus Stent for Acute Coronary Syndrome).^12^

The rationale for our study was to assess the results of these trials. They shared a common aim, that is, to determine after an initial period of treatment with DAPT, whether, as compared with DAPT therapy for 12 months post‐PCI (standard care), time‐constrained treatment with P2Y12 inhibitor monotherapy from 1 to 3 months post‐PCI would be safe and effective with respect to bleeding and MACE.

The trials had, by design, similarities and differences. Considering the timing and duration of study therapy, SMART‐CHOICE, STOPDAPT‐2, TWILIGHT, and TICO involved a strategy of P2Y12 inhibitor monotherapy from 3 to 12 months. GLOBAL LEADERS and STOPDAPT‐2 involved P2Y12 inhibitor monotherapy after 1 month of DAPT, and, unlike the other trials, the duration of P2Y12 inhibitor monotherapy in GLOBAL‐LEADERS was 23 months. The P2Y12 inhibitor medication also varied between the trials. STOPDAPT‐2 involved clopidogrel monotherapy, SMART‐CHOICE involved any P2Y12 inhibitor, and GLOBAL LEADERS, TWILIGHT, and TICO involved ticagrelor monotherapy.

The TWILIGHT and TICO trials had similar designs although there were some important differences. TWILIGHT enrolled 7119 patients (n=4614 [65%] with an ACS) in 187 sites across 11 countries. TICO enrolled 3056 patients with an ACS in 38 centers in Korea. TWILIGHT involved a double‐blind, placebo‐controlled design whereas TICO involved an open‐label design without a placebo. The definitions for major bleeds, for example, Bleeding Academic Research Consortium grades 3 or 5, were broadly similar across the trials.

Our objective was to assess the overall associations in these trials between time‐constrained P2Y12 inhibitor monotherapy (aspirin‐free regimen) for bleeding events, MACE, and all‐cause mortality as compared to standard care with DAPT for up to at least 12 months post‐PCI.

## Methods

We will make the data and methods used in the analysis available to any researcher for the purposes of reproducing the results and procedures.

We undertook a pooled analysis of the data from these trials^8^, ^9^, ^10^, ^11^, ^12^ using DerSimonian and Laird random effects meta‐analysis in the metafor package in R.^13^ The results of the GLOBAL LEADERS trial for post‐PCI participants (though not for ACS subgroup) are presented as relative risk ratios whereas hazard ratios (HRs) are reported in the other trials. The outcomes were primary bleeding outcomes (major and fatal bleeds), MACE, and all‐cause mortality at 12 months. Sensitivity analyses without the GLOBAL LEADERS trial, and with fixed effects meta‐analysis, confirm consistent effects. The analysis followed the Preferred Reporting Items for Systematic Reviews and Meta‐Analyses guidelines (http://www.prism​a-state​ment.org/). Ethical committee review was not required.

## Results

In total, 32 361 patients treated with PCI had outcome data available from 5 randomized, controlled trials of time‐constrained DAPT for 1 to 3 months post‐PCI followed by P2Y12 inhibitor monotherapy versus DAPT for 12 months or longer. The trials (subjects) were GLOBAL‐LEADERS (n=15 968), SMART‐CHOICE (n=2993), STOPDAPT‐2 (n=3045), TWILIGHT (n=7199), and TICO (n=3056). The majority of these patients had undergone PCI following an ACS (n=16 898 [52.2%]).

The main results are shown in Figure. The P2Y12 inhibitor monotherapy strategy was associated with a reduced risk for bleeding post‐PCI (HR, 0.60; 95% CI, 0.45‐0.81) (Figure—Panel A), including in the subgroup of patients presenting with an ACS in whom the magnitude of risk reduction was greatest (HR 0.50; 95% CI, 0.41‐0.61) (Figure—Panel B). The effect was directionally consistent across the trials. P2Y12 inhibitor monotherapy was also associated with reductions in the overall estimates of the hazard of MACE (0.88; 95% CI, 0.77‐1.02) (Figure—Panel C), including in the ACS subgroup (0.86; 0.72‐1.03) (Figure—Panel D), and all‐cause mortality (0.85; 0.71‐1.03) (Figure—Panel E), including in the ACS subgroup (0.73; 0.51‐1.03) (Figure—Panel F), although the upper limit of the CIs marginally exceeded unity.

**Figure 1:**
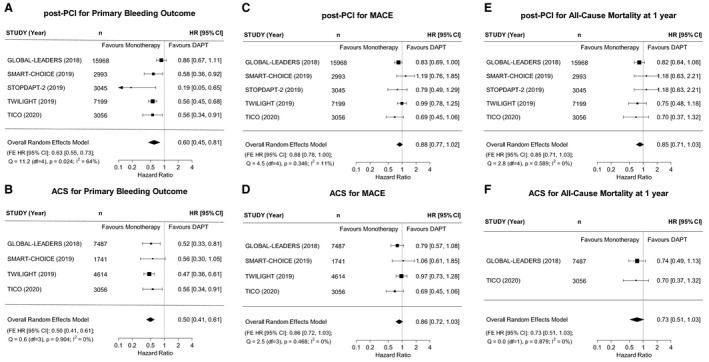
Forest plots for the pooled analyses of the primary bleeding outcomes, major adverse cardiovascular events (MACE), and all‐cause mortality in the 32 361 patients (n=16 898 [52.2%] with a history of acute coronary syndrome [ACS]) enrolled in the GLOBAL LEADERS^8^ (n=15 968), STOPDAPT‐2^9^ (n=3045), SMART‐CHOICE^10^ (n=2993), TWILIGHT^11^ (n=7199), and TICO^12^ (n=3056) clinical trial populations (**A** through **F**). The results are presented for all patients following percutaneous coronary intervention (PCI) and for the subgroup presenting with an ACS for major bleeding outcomes (**A** and **B**), MACE (**C** and **D**), and all‐cause mortality (**E** and **F**), respectively. As well as the overall random effects meta‐analysis hazard ratio (HR) and its 95% CI, fixed effects (FE) analysis results are provided in each of the plots. The random effects and fixed effects meta‐analyses give identical results for most outcomes. Where they differed (in post‐PCI participants for the primary bleeding outcome and MACE), the non‐zero I^2^ indicates the strength of between‐study heterogeneity (Cochran's Q test of heterogeneity is also given). The random effects meta‐analysis should be used in preference, although it should be noted that the differences are small and have no material effect on the conclusions of the study. STOPDAPT‐2 indicates Short and Optimal Duration of Dual Antiplatelet Therapy After Everolimus‐Eluting Cobalt‐Chromium Stent‐2; TICO, Ticagrelor Monotherapy After 3 Months in the Patients Treated With New Generation Sirolimus Stent for Acute Coronary Syndrome; TWILIGHT, Ticagrelor With Aspirin or Alone in High‐Risk Patients After Coronary Intervention.

## Discussion

The pooled results of our analysis indicate that a time‐constrained strategy of P2Y12 inhibitor monotherapy from 1 to 3 months post‐PCI substantially reduces the risk of major bleeding. The effect is greatest in patients with ACS in whom the risk of bleeding is halved.

The purpose for prescribing DAPT is to reduce the risk of MACE. Therefore, antiplatelet monotherapy that is prescribed instead of DAPT within 12 months of receiving PCI might be expected to be associated with an increased the risk of MACE, especially in post‐ACS patients. In fact, reassuringly, we observed a directionally opposite effect. In our analysis, the effect estimates for MACE and all‐cause mortality were less than unity consistent with a lowering of the risks with P2Y12 inhibitor monotherapy. Importantly, considering the worst‐case scenario for the CI, the increase in the HRs was very small. By contrast, the magnitudes of the reductions in the HRs for MACE and all‐cause mortality that were associated with P2Y12 inhibitor monotherapy are substantially greater and consistent with meaningful protective effects. These results are relevant to patient safety. Compared with DAPT for 12 months post‐PCI, P2Y12 inhibitor monotherapy from 1 to 3 months post‐PCI halves the risk of major bleeding and may reduce the risks of MACE and death.

In clinical practice, discontinuation of antiplatelet therapy in patients who are bleeding may lead to MACE, including spontaneous myocardial infarction and stent thrombosis. We have found that prolongation of DAPT to 12 months substantially increases the risk of bleeding. Therefore, the clinical scenario that we describe may be less likely in patients receiving time‐constrained, short‐term DAPT for 1 to 3 months, which may be one explanation for the favorable safety signal for MACE as well as for bleeding. Bleeding and MACE are highly undesirabe events. Considering the net clinical benefit of antiplatelet therapy, bleeding has a very strong association with persisting morbidity and mortality and elderly patients are particularly at risk.^2^ In this regard, practice guidelines recommend a focus on individualized risk (low versus high bleeding risk).^2^ Accordingly, prevention of bleeding should be a primary consideration when prescribing the duration of DAPT in PCI and ACS patients.

The results of our analysis lend support to a strategy of discontinuing aspirin from 3 months post‐PCI and continuing P2Y12 inhibitor monotherapy. Aspirin has been a long‐established, first‐line treatment for secondary prevention of coronary atherosclerosis and a shift to prescribing P2Y12 monotherapy is a new concept for clinicians. Recommendations from practice guideline committees will be important if clinical practice is to evolve in line with the evidence from these trials. The results are also relevant to the safety of participants in ongoing clinical trials comparing DAPT regimens.

Clinicians should still implement individualized therapy decisions for their patients as would normally occur in daily practice. Currently, global prescribing practices for the type of P2Y12 inhibitor are influenced by several factors including access, absolute cost, clinical effectiveness, and practice guidelines.

### Limitations

The risks and benefits of aspirin monotherapy versus P2Y12 inhibitor monotherapy from 1/3 to 12 months following an ACS and/or PCI are not well established. In the future, a large clinical trial will be needed to address this evidence gap.

## Conclusions

P2Y12 inhibitor monotherapy from 1 to 3 months post‐PCI substantially reduces the risk of bleeding without an increase in MACE; rather, reductions in the risks of MACE and all‐cause mortality are evident. There is no evidence of benefit with prolonged DAPT and clear signals harm. Our findings are highly relevant to the safety of patients prescribed DAPT, especially after an ACS. The results support a strategy of discontinuing aspirin from 3 months post‐PCI and continuing with “aspirin free” P2Y12 inhibitor monotherapy in the longer term.

## Sources of Funding

Berry is supported by research funding from the British Heart Foundation (RE/18/6134217; PG/18/52/33892; SP/17/12/32960).

## Disclosures

Berry is employed by the University of Glasgow. which holds research and/or consultancy agreements with AstraZeneca, Abbott Vascular, Boehringer Ingelheim, GSK, HeartFlow, and Novartis. Berry is an investigator and steering committee member in the DUAL‐ACS2 trial (Clini​calTr​ials.gov Identifier: NCT03252249). The remaining authors have no disclosures to report.
